# Chronic Pain and Emotional Stroop: A Systematic Review

**DOI:** 10.3390/jcm11123259

**Published:** 2022-06-07

**Authors:** Lidia Amaro-Díaz, Casandra I. Montoro, Laura R. Fischer-Jbali, Carmen M. Galvez-Sánchez

**Affiliations:** 1 Department of Psychology, University of Jaén, 23071 Jaén, Spain; lamaro@ujaen.es; 2 Institute of Psychology, University of Health Sciences, Medical Informatics and Technology, 6060 Hall in Tirol, Austria; laura.fischer-jbali@umit.at

**Keywords:** chronic pain, emotional Stroop task, brain regions, emotional regulation, attentional bias

## Abstract

Chronic pain is an unpleasant sensory and emotional experience that persists for more than 3 months and is often accompanied by symptoms such as depression, fatigue, sleep disturbances, and cognitive impairment. Emotional dysregulation may also be involved in its etiology. Emotions are known to modulate the experience of pain by influencing cognition and behavior (emotional awareness, emotional expression and experience, and verbalizations). A useful task to explore emotional processing and emotional dysregulation is the emotional Stroop task. Despite the large number of studies using this task, their objectives are diverse; it is necessary to integrate them. The main objective of the present systematic review was to determine the extent of the abnormalities in behavioral performance (including attentional biases) and/or brain alterations in patients with chronic pain during the emotional Stroop task. This systematic review was conducted in accordance with the Cochrane Collaboration guidelines and Preferred Reporting Items for Systematic Reviews and Meta-Analysis (PRISMA) statement. The protocol was previously registered in the Prospective Register of Systematic Reviews (PROSPERO) international database. The selected articles were extracted from the PubMed, Scopus, and Web of Science databases. Fifteen studies were identified as eligible for systematic review. The studies reported alterations in brain regions related to pain and emotional regulation, as well as attentional bias and higher response time latencies (related to the words’ emotional load) in patients with chronic pain. The results confirm the validity of the emotional Stroop task to measure emotions and selective attention. As attentional bias towards negative information is often seen in chronic pain patients, and given the relation between selective attention and greater activation of the brain areas associated with pain and emotional processing, this type of task plays a crucial role in research on emotional and attentional processes among chronic pain patients. Further, attentional bias towards negative information has been associated with higher levels of pain. Taken together, the results suggest the need for cognitive training and an emotional approach to chronic pain therapies, especially targeting attentional biases and negative mood.

## 1. Introduction

### 1.1. Emotional Stroop Task

The emotional Stroop task is a well-established paradigm based on the classic Stroop task [1,2,3]. The aim of this task is to evaluate the interference between emotional stimuli and cognitive processes [4]. Different to the classic Stroop task, in the emotional Stroop task the words presented are emotionally loaded [1,2,5,6]. There are two trial types in both tasks, i.e., incongruent (read the written word and de-code the semantic content; inhibition of an automated action) and congruent (focus on the color of the presented words; activation of a voluntary action) [5,6]. In the emotional Stroop task, the colors of words describing typical chronic pain symptoms (emotionally relevant words) must typically be specified, as well as non-disease-related words with positive, neutral, or negative connotations [2]. These words should be read as quickly as possible, ignoring the affective content of the stimuli presented [7]. This paradigm measures the cognitive interference that occurs when the processing of one stimulus (word) prevents simultaneous processing of a second stimulus (color) [1,8]. According to the emotional Stroop task, the magnitude of the interference effect depends on the extent to which the words are related to the individual’s emotional concerns [1].

The emotional Stroop task is a valuable tool to assess attentional bias in people with chronic pain, and can establish the extent to which patients preferentially attend to pain-related information over neutral or positive information [1,9,10,11]. Therefore, the pain hypervigilance hypothesis pertaining to chronic pain conditions can be investigated by the emotional Stroop task [2,11,12]. This hypothesis suggests that involuntary attention to pain-related information is relevant to the development of these disorders [2,11,12]. Different versions of the task have been applied.

### 1.2. Chronic Pain

Chronic pain is defined by the International Association for the Study of Pain (2020) as a pain condition that lasts for longer than 3 months. It is characterized as a complex sensory and emotional experience that varies according to the context, as well as the meaning of pain, and the psychological state of the individual [13]. Chronic pain has a significant impact on the individual and society [14]. Furthermore, it is considered a standalone condition, rather than a concomitant symptom of other ailments [15]; it causes sleep disruption, depression, and fatigue, as well as limitations in everyday activities and professional work [16]. Furthermore, it is associated with negative emotions and psychological distress [16]. Patients with chronic pain may experience, in certain situations, excessive emotional, cognitive, and behavioral responses [17]. However, the most important clinical symptom of chronic pain is the pain itself [18]. There is a positive correlation between the severity of chronic pain and the intensity of pain and the related phenomenon of outbreaks [18]. Chronic pain has a major impact on the quality of life of those who suffer from it [17,19,20].

Chronic pain is more common in women, elderly people, and the relatively deprived (e.g., those with lower socioeconomic status, disadvantaged geographical and cultural backgrounds, certain employment statuses and occupational factors, or a history of abuse or interpersonal violence) [21]. Several studies of chronic pain reported an inverse relationship between the occurrence of pain and the patient’s socioeconomic status [22,23]. More disadvantaged economic circumstances increase the likelihood of experiencing chronic pain [24]. About 1710 million people have this disease worldwide, including around 20% of the European population [16,21]. The best-known chronic pain diseases are fibromyalgia syndrome (FMS) [2,25,26], migraine [7], temporomandibular disorders (TMDs) [27], chronic musculoskeletal pain (CLBP) [1,28], and chronic neuropathic pain (CNP) [28].

FMS is a chronic widespread pain disorder characterized by generalized musculo-skeletal pain and numerous other symptoms, such as morning stiffness, fatigue, sleep disturbance (insomnia), anxiety, depression, mental decline, cognitive deficits, and reduced health-related quality of life [19,20,29,30,31]. FMS affects about 2–4% of the general population [32,33], with women being more predisposed to it than men [34]. However, the diagnosis of FMS seems to be gender biased, i.e., there is a tendency to overdiagnose FMS in women, even without applying the official criteria [34]. It is thought that overdiagnosis may be mainly due to a lack of knowledge, and a negotiated decision between the patient and doctor to satisfy certain psychosocial needs [34,35]. Although the etiology of FMS is unknown, central sensitization of pain (reflected in hyperalgesia and diffuse allodynia) seems to be the most plausible explanation [36,37]. This is probably due to the fact that FMS involves abnormal processing of pain in the central nervous system and inhibition of antinociceptive inhibitory mechanisms [36,37].

Migraine is an intense pulsing or throbbing pain in one area of the head lasting between 4 and 72 h, and associated with symptoms such as nausea, vomiting, sensitivity to light and sound, preceding neurological symptoms, etc. [38,39]. If migraine persists for more than 15 days a month, for at least 3 consecutive months, it is considered as chronic migraine. Migraine affects 10% of the population, and is more prevalent in women [39,40]. According to Ibrahimi et al. [41], in some women, migraine may be related to changes in hormone levels during the menstrual cycle. Chronic migraine is associated with several comorbidities such as obesity, obstructive sleep apnea, depression, and anxiety, and is also related to excessive use of caffeine and medications (e.g., opioids, barbiturates, and anti-inflammatory drugs) [38]. Pathological neurological and psychological aspects (e.g., a tendency toward perfectionism, rigid and obsessive personality, anxiety, and stress) seem to play a crucial role in the etiology of migraine [39].

TMDs are a group of diseases (temporomandibular joint disorders, masticatory muscle disorders, and disorders affecting associated structures) that affect the oral and maxillofacial region, involve the masticatory muscles and the temporomandibular joint, and can cause chronic pain [42]. The most common symptoms are generalized pain, psychological discomfort, orofacial pain, joint sounds, physical disability, and limitation of mandibular movements [42,43]. The prevalence of this disorder in the general population is between 30–50% [44], and it is more common in women [45]. TMDs have several comorbidities (sleep apnea, migraine, bruxism, neck pain, and biopsychosocial distress) that contribute to the development or persistence of symptoms [46,47]. However, it is not clear whether these comorbidities increase the risk of TMDs or simply coexist with them [48]. Currently, the frequency of somatic symptoms is considered to be the strongest predictor of TMD incidence [48].

Among the different types of chronic pain, CLBP lasts for at least 12 weeks [49], and affects the regions below the costal margin and above the inferior gluteal folds, with or without leg pain [50]. Patients with this disease mainly experience pain in the lower back [50]. Additionally, they exhibit impaired movement and coordination [51]. These disturbances affect the control of voluntary movements [51]. CLBP is the leading cause of disability and the most common of all non-communicable diseases [51,52]. This type of chronic pain has a worldwide prevalence of around 5–10% [16,53]; the prevalence is higher in females, people with less schooling, and smokers [54]. The overall prevalence has doubled over time due to changes in the workplace industry and lifestyles (it is associated with a higher prevalence of obesity, for example) [55]. CLBP is associated with functional cortical, neurochemical, and structural changes in several brain regions, including the somatosensory cortex [56].

CNP can be conceptualized as a pain caused by a lesion or disease of the somatosensory system [57,58]. The painful sensations that accompany CNP (e.g., burning, shooting, tingling, etc.) can be debilitating [59] and long-lasting, even with optimal medical treatment [60,61]. The most common conditions associated with this kind of pain are amputation, leprosy, painful radiculopathy, and trigeminal and postherpetic neuralgia [57]. The most frequent causes of CNP are lumbar and cervical painful radiculopathies [57]. About 6.9–10% of the general population suffers from CNP [21,59,62] and it is more frequent in women [59].

Chronic pain involves physical, psychological, and social factors [15]. The development of chronic pain is associated with risk factors, which are classified as “modifiable” and “non-modifiable” [15]. These include biological, sociodemographic, clinical, and psychological factors [15]. Cognitive and emotional factors strongly influence the connectivity of brain regions that modulate pain perception, emotional states, attention, and expectations [63].

According to imaging studies, the activity of afferent and descendent pain pathways is altered by the attentional state, and by positive and negative emotions [13]. The brain areas most involved in chronic pain are the somatosensory cortex, anterior cingulate gyrus, insula, and the prefrontal and inferior parietal cortices [64]. In addition to these areas, the regions most related to emotions (e.g., the insula, amygdala, and periaqueductal grey) are also involved in this disease [65].

In support of the above, there is considerable evidence of the importance of interventions targeting thoughts, emotions, and behaviors in chronic pain patients [66]. This is due to their associations with distress, the ability to effectively cope with pain, and the perceived intensity of pain [66]. From a physical and psychological point of view, chronic pain is a highly stressful condition that can lead to anger and frustration with both oneself and others [67]. Techniques and therapies concerned with mental and emotional well-being are important to enhance pain resilience [67]. Emotions are involved in the conceptualization, assessment, and treatment of chronic pain [68]. Emotions modulate the experience of pain by influencing cognitions and behaviors (emotional awareness, emotional expression and experience, and verbalizations) [68].

### 1.3. Previous Reviews on the Emotional Stroop Task and Chronic Pain

According to the reviewed literature, and as previously reported, the emotional Stroop task is a valuable and suitable technique to measure the alterations in emotional and cerebral activation areas that characterize chronic pain conditions (i.e., FMS, migraine, CNP, CLBP, and TMDs) [2,9,69]. Other reviews related to chronic pain and the emotional Stroop task assessed the attentional bias of patients with chronic pain [11,70], as well as the origins thereof [71]. However, each review used the emotional Stroop task for different objectives, such as to characterize cognitive inhibition mechanisms and attentional control functions in patients with FMS [25], assess attentional biases for negative affective stimuli related to migraine [7], test the hypothesis of generalized hypervigilance in FMS and explore the possible mediating role of anxiety [26], and investigate attentional bias in patients with chronic pain [9,70]. Furthermore, it seems that findings related to the emotional Stroop task and chronic pain are equivocal. Although in the majority of studies attentional bias in people with chronic pain was demonstrated [1,2,11], other studies, such as Andersson et al. [9], did not observe significant effects in terms of inhibition or increased interference during color naming in chronic pain.

Given the modulatory effect of emotions on pain, the importance of exploring and integrating all of the previous results, especially those addressing pain and emotional processing, should not be overlooked. Accordingly, the main objective of the present systematic review was, for the first time, to perform an integrated analysis of all studies using an emotional Stroop task to assess the associations of alterations in specific brain regions with the behavioral performance (e.g., attentional biases) of patients with chronic pain.

## 2. Materials and Methods

### 2.1. Search Strategy

This systematic review was conducted based on the guidelines of the Cochrane Collaboration, and in accordance with the Preferred Reporting Items for Systematic Reviews and Meta-Analyses (PRISMA) [72]. As a first step, the inclusion and exclusion criteria, as well as the analyses, were specified. Subsequently, the protocol was registered in the Prospective Register of Systematic Reviews (PROSPERO) international database (Registration ID: CRD42021279615). The following terms, extracted by MeSH (Medical Subject Headings), were used for the search: chronic pain and emotional Stroop. The last search was carried out on 1 March 2022.

**Figure 1:**
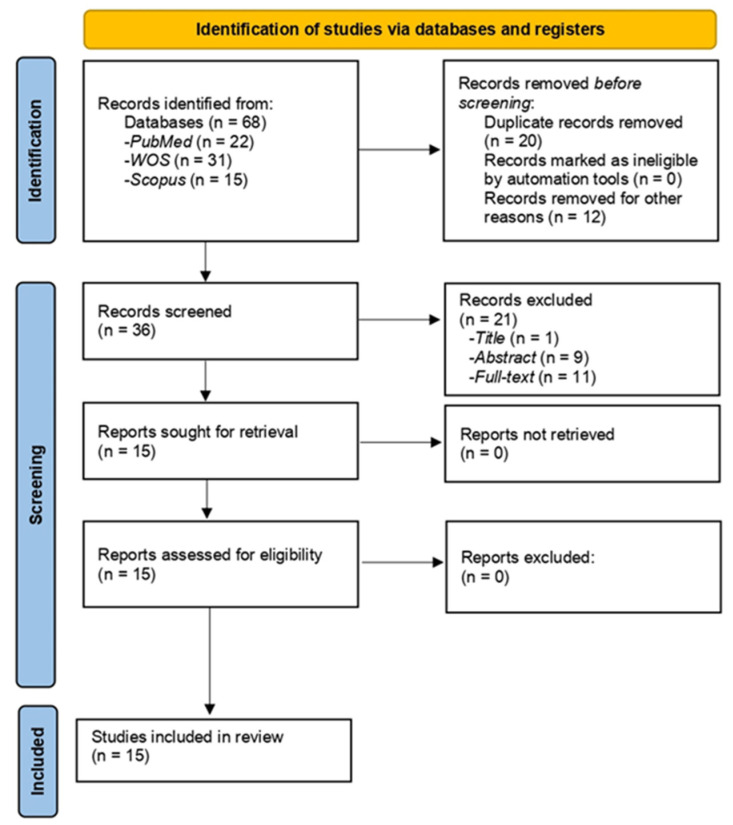
Flow diagram of Chronic Pain and Emotional Stroop (PRISMA).

Independent searches of the Scopus, PubMed, and Web of Science (WOS) databases were conducted by three researchers (L.A.-D., C.I.M.-A., and L.R.F.-J.). All of the identified articles were reviewed, and those that did not meet the criteria for subsequent analysis of the full text were discarded. First, in order to eliminate irrelevant studies, the titles and abstracts of each study were analyzed. In a second step, the remaining articles were screened in detail for eligibility. All full texts of the selected articles were checked and analyzed based on the inclusion and exclusion criteria. Any discrepancies found during the review of these articles were reviewed by the fourth author (C.M.G.-S.). The PRISMA flowchart (Figure 1) shows the screening and selection process for the inclusion of studies. In addition, C.M.G.-S. examined all articles for eligibility for the study prior to data extraction and quality assessment. The PICO question was as follows: How do patients with chronic pain perform in the emotional Stroop task?

### 2.2. Eligibility Criteria

The following study inclusion criteria were applied: (1) written in English or Spanish; (2) original, peer-reviewed study; (3) adult patients (≥18 years old); and (4) focused on chronic pain and the emotional Stroop task.

The exclusion criteria were as follows: (1) written in languages other than English or Spanish; (2) review article or meta-analysis; (3) inclusion of non-adult patients (≤18 years old); and (4) commentary, report, letter, editorial, meeting, and/or congress abstract or case report.

### 2.3. Data Extraction and Quality Assessment

L.A.-D., C.I.M.-A., and L.R.F.-J. independently extracted study characteristics, methodologies and results, and assessed the limitations of each study. Discrepancies were reviewed by C.M.G.-S. The sequence for data extraction was as follows: first author, study name, country, year of publication, study design, sample size, number of participants in each study group, and age and sex of the participants. The characteristics of the study are shown in Table 1. C.M.G.-S. reviewed all data to ensure the accuracy of the data extraction.

### 2.4. Data Synthesis

Our review focuses on studies of patients over 18 years of age, suffering from any chronic pain condition (FMS, CLBP, CNP, migraine, or TMD). In addition, all of the studies used the emotional Stroop task (or any variant thereof), and the performance of patients with chronic pain was compared (in most cases) with a control group composed by healthy participants. Attention was also paid to the activation of brain areas responsible for emotional and pain processing during the application of the emotional Stroop task, as well as to possible attentional biases towards certain types of stimuli presented in the task.

According to the objectives of this review, the type of the Stroop task performed in each study (and whether the sample had a control group) and the study design (e.g., randomized controlled trial, experimental, or case-control study) were determined. In addition, the target population, as well as the proportion of male and female participants, and their mean and standard deviation age, were ascertained. Furthermore, the main results of each study, which are shown in Table 1 (first author, study name and country, objective, diagnostic technique, sample size and age, type of emotional Stroop task, and results), were analyzed. Finally, the limitations of each study were assessed.

## 3. Results

### 3.1. Literature Search and Study Characteristics

After a comprehensive search, 68 relevant articles were identified in the databases. After eliminating duplicates, a total of 36 articles were selected for this review. The PRISMA flow diagram (for details, see Section 2.1. Search strategy) shows the exclusion of studies at each screening stage (Figure 1). Finally, 15 full-text articles were included; they were checked for suitability according to the predefined inclusion criteria, and then subjected to data extraction (Table 1) and quality assessment.

Regarding the characteristics of the selected studies, the year of publication ranged from 1989 to 2021. Most of the studies included a control group of healthy participants [2,7,9,25,26,28,73,74,75,76,77,78], although two were uncontrolled clinical trials [1,28]. The location of the studies varied widely: 11 were conducted in Europe (Spain [25,26], United Kingdom [69,76,78], Sweden [9], Germany [7], Austria [2], Netherlands [28], and Belgium [1]), 1 in the United States [77], and 4 in Canada [27,73,74,75]. Further details of the characteristics of the selected studies can be found in Table 1.

In terms of the study designs, an experimental design was used in the majority of cases [1,2,25,26,27,28,69,74,75,77,78], although two studies used a factorial design [9,76], one used a case-control design [7], and one used a pilot randomized controlled trial design [73].

Regarding the results of the studies (Table 1) related to performance on the emotional Stroop task, longer reaction times and delayed responses to negative emotional words (associated with pain) were observed in patients with chronic pain, especially those with FMS [7,9,25,26,74,75,76,77,78]. Most studies revealed an emotional interference effect in FMS patients [25,79,80]. Greater processing of negative and/or positive words was also observed in patients with FMS [79,81]. Moreover, greater responses were observed in regions related to pain and emotional regulation (the somatosensory region, and the cingulate and prefrontal cortices, among other regions) compared to healthy controls [27,69,73]. Specifically, in the presence of negative stimuli with emotional content, patients with chronic pain showed greater activation in the aforementioned brain regions, indicating greater processing of pain and negative emotions in these patients [27,69,73]. Previous evidence indicates that patients with CLBP and FMS have an attentional bias towards negative words with emotional content [1,2,9,74].

### 3.2. Participants

Among the 15 selected articles, 7 used the Stroop task for patients with general chronic pain [9,69,74,75,76,77,78], while 3 used it for FMS patients [2,25,26], 2 for CLBP patients [1,28], 1 for migraine patients [7], 1 for TMD patients [27], and 1 for CNP patients [73].

The total study sample (*n* = 677) was divided into two main groups: a clinical group (*n* = 386) and a control group (with slightly fewer participants; *n* = 291). The clinical group included 11 women with CNP (age range: 48–57 years) [73] and 17 with TMDs (age range: 34–36 years) [27], 17 participants with migraine (85% women) [7], 19 women, 11 men and 25 participants of unspecified gender with CLBP (age range: 35–49 years) [69,74], 77 women with FMS (age range: 47–54 years) [2,25,26], 33 participants of unspecified gender with chronic back and/or neck pain (age range: 30–36 years), and 94 women, 50 men and 33 participants of unspecified gender with general chronic pain (age range: 38–83 years) [9,69,74,75,76,77,78].

It should be noted that two of the studies did not have a control group [1,28]. Regarding subjects’ sex, there were more female than male participants [2,7,9,25,26,27,28,73,74,76]. Nevertheless, five studies included both men and women [7,9,28,74,76]. Notably, none of the reviewed studies included a sample composed entirely of men. Further, five studies did not provide information about the sex of the participants [1,69,75,77,78].

In the selected articles, the majority of the chronic pain participants did not have comorbid psychiatric illnesses (e.g., depression or anxiety) [1,7,9,25,26,27,28,74,75,77]. Furthermore, some of them used these conditions as exclusion criteria [7,25,26,27,69,74,76,77]. Other studies considered these conditions as a symptom of chronic pain, especially in FMS [2,25]. However, these conditions could negatively influence emotional Stroop task responses [76,82].

### 3.3. Quality of Selected Studies

The quality assessment was conducted independently by two researchers (L.A.-D. and C.I.M.-A.), and the initial agreement was 93%. To achieve a consensus, the interpretation and monitoring of the criteria was discussed with a third reviewer (C.M.S.-G.). This quality assessment was focused on the analysis of the limitations of the selected studies.

The authors of the reviewed studies indicated various limitations of their research, such as small sample and effect sizes [2,7,9,26,73,74,75,77], an absence of a neutral category of words [28], issues with the methods and/or criteria used for the diagnosis of chronic pain (e.g., ACR criteria for FMS and IHS criteria for migraine) [2,75], and with pharmacological treatments [2,25,69,75], non-control of the medication status (pharmacological) of the patients and healthy controls [75], low statistical power and non-inclusion of an additional experimental condition for the Stroop task [26], non-inclusion of a masked version of the test and failure to record each participant’s pain level at the time of the test [74], use of non-specific stimuli [1,7,9,28,73,74], non-inclusion of additional measures of psychological distress to improve construct validity [77], a non-pragmatic approach to the study patients [69], failure to assess sensitivity to anxiety or fear of pain, failure to screen for psychiatric disturbances through screening interviews, and non-inclusion of a pain comparison group [9], and failure to control for the effect of some variables (e.g., time since surgery, dose of chemotherapy received, type of chemotherapy drugs, current medications and menopausal) [73].

Additional limitations were identified during this review, including non-randomization of participants to different groups in most studies [1,2,7,9,25,26,27,28,69,74,75,76,77,78], the absence of a control group to compare the results [1,28], non-blinding of participants, personnel and outcome assessments [1,2,7,9,25,26,27,28,69,74,75,76,77,78], non-specification of the criteria used to diagnose the disease [69,73,74,77], failure to report effect size measures [1,7,25,27,28,69,73,75,76,77,78], failure to indicate the sex ratio in some studies [1,69,75,77,78], and failure to report analyses by sex [69,76]. Moreover, some studies did not report the mean age [7,69,76] or standard deviation of their sample [69,76], and provided incomplete data on the task performed (e.g., failure to disclose the total duration of the task) [1,7,9,27,28,69,77].

## 4. Discussion

The present systematic review aimed to analyze studies that used an emotional Stroop task in patients with chronic pain, and assessed associated alterations of specific brain regions and behavioral performance (e.g., attentional biases). In general, and as reported in the literature, the emotional Stroop task proved to be a valid tool to assess emotional and pain processing in patients with chronic pain [2,9,69]. Most studies reported the activation of certain brain regions (the somatosensory region, and cingulate and prefrontal cortices, among other regions) during the emotional Stroop task; these regions are related to pain and emotional regulation in patients with chronic pain [27,69,73].

First, patients’ performance in the emotional Stroop task, as well as the presence of attentional biases, will be discussed, followed by a brief overview of the brain areas showing neural activation in relation to the performance of the emotional Stroop task. Finally, the benefits and effects of psychological therapies that can reduce the neural activation observed in patients with chronic pain will be discussed.

Regarding performance on the emotional Stroop task, greater processing of negative and/or positive words was observed in patients with FMS, suggesting the existence of an underlying interference process, triggered by events capable of immediately capturing attention (i.e., those conveying affective meaning) [78,81]. Studies such as that of Algom et al. [83] indicate that this interference effect in the emotional Stroop task is mediated by pre-attentive inhibition, associated with the threat of negative emotional stimuli presented during the task. However, this inhibition mechanism is considered to be independent from that of selective attention [83]. In FMS patients, delayed responses to pain words were associated with pain-specific anxiety and cognitive interference, as well as low sensitivity to anxiety [75]. Some studies indicated that the slowness in color naming during the emotional Stroop task seen in FMS patients is associated with the presence of a generalized hypervigilance response [12,26]. This response is associated with a tendency for FMS subjects to be slower with respect to the color naming of symptomatic (pain-related words) and arousing negative words, depending on the degree of perceived unpleasantness [9,26]. However, it is suggested that, in larger samples, more significant interactions between patient and control groups would be seen, and that it is necessary to compare these findings with those for other diseases [9,12,26,69]. In patients with CLBP and FMS, attentional bias to sensory pain words was associated with the emotional load of the words presented in the emotional Stroop task [1,2,9,74]. This provides clear evidence of the presence of emotion-driven selective attention in FMS and CLBP [1,2,30,84]. In fact, the existence of attentional bias towards negative information seems to play an important mediating role in the relationship between a negative affective state and heightened pain [2,30,84]. In the study by Duschek et al. [2], such attentional bias was also observed in patients with FMS; they showed a specific bias towards negative information, which led to an increase in pain intensity. This further supports the findings of the literature reviewed herein. In CLBP, attentional bias was even greater in the context of words related to back pathology, and in association with increased pain intensity [1,76]. However, the causal nature of the relationship between attentional bias and pain could not be established, as most of the included studies used a cross-sectional design. On the other hand, there are data showing that individuals with greater attentional bias towards negative affective stimuli (i.e., words associated with pain) may be more prone to chronic pain symptoms [85]. In fact, attentional bias in these individuals may be a risk factor for the development of chronic pain and could also serve as a prognostic factor [71]. Attentional bias has been consistently linked to individuals’ anticipation and/or experience of pain across different chronic pain conditions [70,85].

In terms of neuronal activation, in patients with chronic pain in general, greater activation was observed when performing the emotional Stroop task [69]. Compared to the healthy group, greater activation in the anterior cingulate cortex, insula, and the primary and secondary somatosensory cortex was seen [69]. More specifically, pain-related words in the Stroop task were associated with significant differences between chronic pain patients and healthy controls, in terms of activation of the pain-processing centers of the brain (i.e., the anterior cingulate cortex, insula, parietal operculum, and the primary and secondary somatosensory cortices) [11,69]. Greater activation of brain areas related to attention, cognition, and motor planning in patients with TMDs compared to controls was also found [27]. TMD subjects showed increased task-evoked responses in prefrontal, lateral, and inferior parietal areas, as well as in the amygdala, pregenual anterior cingulate, primary motor areas, and the medial prefrontal and posterior cingulate areas [27,86]. In addition, patients also showed dissociations with respect to the activity of the prefrontal cortex and cingulate, and of the amygdala and cingulate, which are normally correlated [27,86,87,88]. Hence, the prominence of chronic pain (which requires attention) and slow behavioral responses may be explained by attenuated, or slow and/or desynchronized, recruitment of attentional processing areas [27,86,87,88].

Some of the studies reviewed herein focused on specific psychological therapies, such as the mindfulness-based stress reduction technique, which yielded a significant reduction in brain activity in regions related to pain, emotional regulation, and cognitive processing (i.e., regions in the left somatosensory cortex, left precuneus, and left dorsolateral prefrontal cortex) in patients with CNP, using the emotional Stroop task as a measure of emotional reactivity [73]. This demonstrates the impact of psychological therapy on the neural correlates of pain processing and attention [73]. Mindfulness-based psychological therapies seem to be a viable complementary treatment for people suffering from CNP [73,89]. Indeed, the reduction in cerebral activity observed after mindfulness treatment suggested that the emotionally charged words presented during the task had a diminished capacity to capture attention after the therapy compared to before the therapy [90,91]. Thus, the application of this technique reduces brain activation and pain perception, where trait mindfulness is a major component of the therapy [90,91]. However, to draw firm conclusions, longitudinal studies regarding the effect of this type of psychological therapy on patients with chronic pain are needed. Moreover, other therapies, such as cognitive behavioral therapy, have been used in patients with TMDs to effectively reduce the abnormal neuronal and brain activation seen in patients after performing the emotional Stroop task [27]. Likewise, in patients with FMS, cognitive therapy for chronic pain has focused on reducing the negative attentional bias exhibited by these patients [2]. The self-control strategies involved in this therapy promote conscious withdrawal of attention from dysfunctional cognitions and possible stressors, such as emotionally charged negative words, after the application of the Emotional Stroop task [2]. Similarly, techniques such as attention training, focusing, and exposure (cognitive behavioral therapy) have proven useful in patients with FMS, to reduce the activation of emotional and pain processing areas after the application of the emotional Stroop task [26]. This therapy also reduces the hypervigilance exhibited by patients with FMS, and attentional bias to negative emotions [26].

An important limitation of the present review is that the majority of the sample was female [2,7,9,25,26,27,28,73,74,76], where the overall gender ratio of the studies was not equal. However, as previously noted, chronic pain is more prevalent in females, in whom it also tends to be overdiagnosed [34], and so studies frequently include a larger female sample. Another limitation is the lack of information on effect sizes [2,7,9,26,73,74,75,77]; this lack of information on the magnitude of the differences found limited the interpretability of the results. In addition, some of the studies did not specify the clinical criteria used to diagnose the different types of chronic pain [2,75], which calls into question whether the diagnoses were made on the basis of valid criteria. Furthermore, only one of the selected studies did not obtain statistically significant results [77]. A possible explanation for this may be insufficient sample sizes, which tend to preclude large variability in the results. In addition, a more accurate study quality assessment tool will be necessary for future studies. To further elaborate on the results obtained by each study, future reviews could perform a meta-analysis and also compare the findings with those of other emotional tasks, such as the dot-probe or spatial cueing task. Finally, to overcome the absence of effect sizes in the studies [1,7,25,27,28,69,73,74,75,76,77,78], calculation (and pooling) of Cohen’s d or standardized measures of means would be useful (although this is more crucial and typical for meta-analyses).

The main strength of the present review was that it strictly followed a systematic methodological approach in accordance with the study protocol, which was previously registered in PROSPERO, and was prepared in accordance with the updated PRISMA guidelines [72]. Further, in terms of the thematic focus of the systematic review, this is the first review to relate the emotional Stroop task to chronic pain.

In light of the findings of the present review and the analyzed literature, continuing to examine the efficacy of the emotional Stroop task in patients with chronic pain is of high clinical relevance. Future research on chronic pain and the emotional Stroop task should aim to uncover neurobiological correlates in chronic pain patients during performance of the task. Once the precise neuroanatomical correlates underlying the disease are known, specific and integrated research and/or intervention protocols can be established to improve health-related quality of life. Given the negative attentional biases that chronic pain patients exhibit during the performance of the emotional Stroop task, a treatment aimed at the conscious redirection of attention against negative aspects could be implemented, along with relaxation techniques, modification of beliefs about pain, enhancement of coping skills, and targeted treatment of anxiety and/or depression [66]. Future research aiming to establish a relationship between attentional bias and the anticipation and/or experience of pain would be also useful to identify individuals at risk of developing chronic pain, as well as prognostic factors. Psychological treatments can be as effective as surgery for alleviating chronic pain symptoms, by altering the central processing of pain sensation [66]. In this sense, another therapy suitable for chronic pain patients is Acceptance and Commitment Therapy (ACT), the mindfulness component of which is the basis of mindfulness therapy. This therapy has proven effective for people with chronic pain [92,93,94,95]. In addition, chronic pain patients with a history of psychosocial trauma may benefit from exposure and emotional processing techniques, which have proven effective [96]. Following the application of the emotional Stroop task, studies suggest that the use of other psychological therapies may be beneficial in reducing brain activation (e.g., the cingulate, amygdala, and medial prefrontal cortex) [2,26,27]. Specifically, Weissman-Fogel et al. [27] suggested that cognitive behavioral therapy could be effective for reducing brain activation after the application of the emotional Stroop task in patients with TMDs. Unfortunately, there are currently no studies that have evaluated the effectiveness of the aforementioned techniques in reducing activation in these areas (i.e., the cingulate, amygdala, and medial prefrontal cortex). Nonetheless, such therapy (e.g., self-control, focusing, exposure, and attentional training) and cognitive therapy in patients with FMS can reduce hypervigilance towards negative stimuli with emotional content [2,26].

Numerous models of the development and/or maintenance of chronic pain suggest that attentional biases are important therein [97,98]. These models support the findings of the present systematic review. Furthermore, each model attributes different roles to attentional processes [97,98]. However, they all make the same assumption; people pay excessive attention to painful stimuli when they experience pain and feel fearful of, or threatened by, pain [97,99]. Indeed, as it was mentioned above, there is an attentional bias towards negative stimuli in people with chronic pain [2]. Furthermore, increased attention to threat/negative cues has been observed in patients with other conditions apart from chronic pain (e.g., anxiety disorders and post-traumatic stress disorder) [9,73]. This bias might be explained by the Threat Interpretation Model [100], which suggests a relationship between threat, threat interpretation, and stimuli, through the vigilance–avoidance hypothesis [100]. This hypothesis states that individuals usually pay more attention to threat stimuli and this attentional bias is usually accompanied by an avoidance of negative/threat stimuli [100]. This pattern of vigilance–avoidance may be variable across individuals, as the interpretation of stimuli may differ according to the degree of importance assigned to the perceived stimuli [100]. Furthermore, this model may generate verifiable predictions about the role of attentional processes, and how they are influenced by interpretations [100], which would have a positive impact on clinical practice (i.e., the development and improvement of chronic pain treatments).

In conclusion, after performing the emotional Stroop task, specific brain areas (e.g., the prefrontal cortex, somatosensory cortex, cingulum, and amygdala) related to emotional and pain processing are activated in patients with chronic pain (FMS, migraine, CNP, TMDS, and CLBP). During the task, chronic pain patients showed longer reaction times and delayed responses to words with negative emotional content. They also showed attentional biases towards pain sensory words. Therefore, the use of psychological therapies (e.g., mindfulness, cognitive, and cognitive behavioral therapies) will help reduce the brain activation and attentional bias produced by the emotional Stroop task in these patients.

## Figures and Tables

**Table 1:** Characteristics of selected studies on Chronic Pain and Emotional Stroop.

Chronic Pain and Emotional Stroop					
First Author (Publication Year), Study Name, Country	Objective	Study Design/Diagnostic Technique	Sample Size, Age (Mean ± SD)	Emotional Stroop	Results
Hatchard et al. [73] Reduced emotional reactivity in breast cancer survivors with chronic neuropathic pain following mindfulness-based stress reduction (MBSR): an fMRI pilot investigation. Canada.	To analyze the impact of MBSR on the emotional reactivity of breast cancer survivors with CNP (8 weeks).	Randomized controlled trial (pilot study). MBSR program: pain disability, psychological well-being, and overall quality of life.	N = 21 women. MBSR treatment group = 11 (48.36 ± 11.37). WL control group = 10 (56.50 ± 8.11).	Modified Stroop task. Stimuli: 8 blocks (each block 16 words and 33 s long): 4 pain-related sensory and negative affective words and 4 neutral words. Total duration: 7 min.	MBSR treatment group: less BOLD activity post-MBSR across several brain regions (pain processing and visual attention). Reduced pain interference following MBSR.
Taylor et al. [69] Neural responses to a modified Stroop paradigm in patients with complex chronic musculoskeletal pain compared to matched controls: an experimental functional magnetic resonance imaging study. United Kingdom *.	To investigate the general deficit in attentional control and specific attentional bias for pain-related stimuli and BOLD signal differences in pain and emotion related brain regions.	Experimental fMRI study. Patients with CMSKP: BOLD fMRI.	N = 29 (25–83 years). CMSKP group = 15. HC group = 14.	Modified Stroop task. Stimuli (16 from each word group): pain-related, positive-emotional, and neutral control words. 16 blocks: 2 word-type, 2 control word set, and 4 fixation-cross (the rest in each run). Total duration: less than 15 min (with a short break).	CMSKP group: less accurate in responses (all word types). BOLD fMRI responses: increases in neural activation in CMSKP group (anterior cingulate cortex, insula, and primary and secondary somatosensory cortex).
Duschek et al. [2] Attentional bias toward negative information in patients with fibromyalgia syndrome. Austria.	To investigate the contribution of specific features (FMS) to expected attentional bias.	Experimental study. FMS diagnosis: ACR (Wolfe et al., 1990).	N = 61 women. FMS group = 27 (52.70 ± 9.20). HC group = 34 (53.90 ± 8.40).	Modified emotional Stroop task. Stimuli: 40 positive, 40 negative, and 40 neutral adjectives. Color word: below adjective printed in black (500 ms after stimuli) **.	FMS: attentional bias, delayed response (negative words), and reaction times longer (negative words). Association with interference scores for positive and negative words (severity of FMS and attentional bias toward affectively negative information).
Mercado et al. [25] Brain correlates of cognitive inhibition in fibromyalgia: emotional intrusion of symptom-related words. Spain.	To characterize cognitive inhibition mechanisms and attentional control functions (FMS).	Experimental study. FMS diagnosis: ACR (Wolfe et al., 1990).	N = 50 women. FMS group = 25 (47.80 ± 8.34). HC group = 25 (48.00 ± 7.48).	An emotional variant of the Stroop task. Stimuli (4 categories): linguistic words (300 ms); FMS SF, arousing; negative and positive; and neutral (32 words: red, blue, yellow, and green) **.	SF words: part of their own symptoms (FMS). RT: faster in SF words. Number of errors: smaller for SF words.
Puschmann et al. [7] Hypervigilance or avoidance of trigger related cues in migraineurs?—A case-control study using the emotional stroop task. Germany.	To assess attentional biases for negative affective stimuli related to migraine.	Case-control study. Migraine diagnosis: IHS (2004).	N = 53. EM group = 17 (41.35 ± 11.87) (85% women). FM group = 16 women (43.40 ± 13.30). HC group = 20 (39.80 ± 10.50) (90.5% women).	Computerized version of the modified emotional Stroop task. Task 1: General affective words: 36 nouns (Berlin Affective Word List); 12 for each valence (negative, neutral, and positive). Time words: 2000 ms. Time between words: 500 ms **. Task 2: 81 affective face pictures (Karolinska Directed Emotional Faces). 3 stimuli (each category 27 pictures): positive, negative, and neutral. Maximum response: 2000 ms **.	FM group: responded faster to negative stimuli and learned avoidance mechanism away from affective migraine triggers.
Weissman-Fogel et al. [27] Abnormal cortical activity in patients with temporomandibular disorder evoked by cognitive and emotional tasks. Canada.	To test if patients with TMD perform poorly in cognitive and emotion tasks and abnormal task-evoked brain activity.	Experimental study. TMD diagnosis: specialist dentists (Pain Unit of the Mount Sinai Hospital Dental Clinic), standard clinical criteria, and involvement of myofascial and/or temporomandibular joint (clinical testing).	N = 34 women. TMD group = 17 (35.20 ± 11.6). HC group = 17 (34.00 ± 9.90).	nStroop: common household items. ncStroop: number words (cognitive interference). ecStroop: TMD-related emotional words (emotional interference). Stroop block: 12 sets of words (1250 ms) **.	Each Stroop task activated brain areas (attention, cognition, and motor planning). TMD patients: sluggish reaction times for all Stroop tasks and decoupling of the normally positively associated activity between prefrontal—cingulate cortices and between amygdala—cingulate cortex.
González et al. [26] Generalized hypervigilance in fibromyalgia patients: an experimental analysis with the emotional Stroop paradigm. Spain.	To test the hypothesis of generalized hypervigilance in FMS and explore the possible mediating role of anxiety.	Experimental analysis. FMS diagnosis: (Wolfe, Smith, Yunus et al.).	N = 50 women. Final sample = 49. FMS group = 25 (50.56 ± 8.66). HC group = 24 (48.04 ± 7.55).	Emotional Stroop task. 4 stimuli (32 words): fibromyalgia symptoms and neutral, positive, and negative arousal. Stimuli: 300 ms (interval 1.5–2 s) **. 4 colors: red, green, yellow, and blue. 32 trials (128 randomized trials).	Possible presence of generalized hypervigilance response in FMS patients (significant slowness in the color naming).
Asmundson et al. [74] Hypervigilance and attentional fixedness in chronic musculoskeletal pain: consistency of findings across modified stroop and dot-probe tasks. Canada.	To investigate attentional biases for sensory and affect pain stimuli in CMSKP patients.	Experimental analysis. CMSKP diagnosis: (rehabilitation program—Regina urban area).	N = 75. Final sample = 65. CMSKP group = 36 (women = 22 (36.27 ± 11.76); men = 14 (40.79 ± 9.38)). HC group = 29 (women = 18 (42.00 ± 10.64; men = 11 (35.91 ± 10.30)).	Computerized modified Stroop task. Stimuli: 15 sensory pain, 15 health catastrophe and 15 neutral words. 4 colors: red, blue, yellow, and green. 10 blocks: 30 trials per block **.	CMSKP group: initial attention to the threat positively associated with vigilance for that particular threat, and negatively associated with disengagement from the threat.
Roelofs et al. [28] An examination of word relevance in a modified stroop task in patients with chronic low back pain. Netherlands.	To examine the role of personal relevance of sensory pain-related words in selective attentional processing in low back pain patients.	Experimental study. Chronic low back pain diagnosis: Belgian pain clinics (University of Ghent and University Hospital of Leuven at ‘Pellenberg’).	N = 30. CLBP group = 30 (41.20 ± 11.60) (19 women).	Computerized version of modified Stroop task. Stimuli: 33 sensory pain-related words. Colors: red, blue, yellow, and green. 132 trials. Total duration: 8 min.	No significant results. No support for the hypothesis that sensory pain-related words interact with Fear of Pain scores in accounting for reaction times (naming the color of sensory pain-related words). Modified Stroop task not a robust measure of selective attentional processing in chronic low back pain patients.
Andersson et al. [9] Personalized pain words and Stroop interference in chronic pain patients. Sweden.	To investigate attentional bias in patients with chronic pain.	Mixed design. One between-group factor and one within-group factor in a 2 × 2 design. Chronic pain diagnosis: local pain clinic.	N = 40. Chronic pain group = 20 (44.50 ± 9.82) (16 women). HC group = 20 (45.60 ± 9.45) (16 women).	Computerized modified version of emotional Stroop task with personalized words. 6 trials: Color-naming pain and control words (99 words each trial). 5 colors: blue, red, yellow, white, and green. Total duration: 15–23 min.	Pain group: slower on pain words and longer on color-name pain words. 11 chronic pain patients: Stroop interference effect. Repeated measure effect: threat word category and Stroop color naming.
Snider et al. [75] Automatic and strategic processing of threat cues in patients with chronic pain: a modified stroop evaluation. Canada.	To determine if chronic back and/or neck pain patients exhibit delayed color-naming latencies for syndrome-specific cues (strategic and automatic levels of processing).	Experimental study. Back and/or neck pain diagnosis (minimum 3 months). Rehabilitation program in Regina Health District.	N = 66. Chronic back and/or neck pain group = 33 (35.50 ± 10.30). HC = 33 (35.00 ± 10.10).	A modified Stroop evaluation. Stimuli: 10 affect pain, 10 physical threat, 10 social threat, and 10 neutral words. 200 trials. 50 words, 2 times: unmasked and masked conditions. 4 colors: red, blue, yellow, and green. 10 blocks: 5 unmasked and 5 masked (20 trials for each one) **.	Chronic pain patients: selectively process pain-related cues at the strategic level. Delayed color-naming latencies (sensory and affect pain words; unmasked condition). Delayed color-naming latencies (pain words; unmasked condition) positively associated with high pain-specific cognitive anxiety and interference and lower levels of anxiety sensitivity.
Crombez et al. [1] The emotional stroop task and chronic pain: what is threatening for chronic pain sufferers? Belgium.	To investigate chronic pain patients display an involuntary attentional shift towards pain-related information.	Experimental study. CLBP diagnosis: pain clinic —physical rehabilitation unit (university clinic).	N = 25. CLBP group = 25 (48.36 ± 14.12).	Computer version of emotional Stroop task. 5 experimental stimuli: 7 sensory pain, 7 affect pain, 7 related back disorder, 7 other disorder, and 7 general negative valence words (5 neutral words in each category). Total words: 70 **. 4 colors: blue, yellow, green, and red.	Attentional bias: sensory pain words. Current pain intensity predictive of the effect.
Pincus et al. [76] Do chronic pain patients ‘stroop’ on pain stimuli? United Kingdom *.	To investigate the presence of information processing biases on tasks of attention and memory in relation to mood states in chronic pain patients.	Experiment 1: 2 × 4 factorial design. Chronic pain diagnosis: hospital pain clinic. Experiment 2: 2 × 8 factorial design. Chronic pain diagnosis: hospital pain clinic.	Experiment 1: N = 40. Chronic pain group = 20 (18 women). HC group = 20 (12 women). Experiment 2: N = 34. Chronic pain group = 17 (12 women). HC group = 17 (11 women).	Experiment 1: classical Stroop and congruent color naming. 10 blocks. Stimuli (10 words for each one): sensory, affective, positive, and neutral words. 5 colors: red, brown, blue, orange, and green. 50 trials **. Experiment 2: classical stroop, color naming. Stimuli (10 words for each one): sensory, affective, positive, physical threat, social threat, and household objects. 50 trials. 3 colors: pink, yellow, and green. Interval between words: 500 ms **.	Memory recall bias. Interference effect for emotionally salient stimuli related to anxiety and depression.
Duckworth et al. [77] Information processing in chronic pain disorder: a preliminary analysis. United States of America.	To establish the comparative usefulness of selective attention, impaired stimulus filtering, and affective language deficiency models for explaining somatic focus in a chronic pain population.	Experimental study. Chronic pain diagnosis: interdisciplinary facility (Athens, Georgia).	N = 29. HSF group = 10 (43.10 ± 12.00). LSF group = 9 (38.20 ± 12.20). HC group = 10 (39.30 ± 11.10).	Modified Stroop task. Stimuli (105 words): 35 somatic pain-content, 35 depression-content, and 35 neutral-content (5 s for each one). 5 colors: red, yellow, green, blue, and white. Total duration: 15 min.	Chronic pain patients misinterpret bodily sensations.
Pearce et al. [78] An experimental investigation of the construct validity of the McGill Pain Questionnaire. United Kingdom.	To avoid problems with self-report measures of pain.	Experimental study. Chronic pain diagnosis: pain clinic.	N = 32. Chronic pain group = 16 (53.50 ± 14.10). HC group = 16 (52.60 ± 14.50).	Stroop task. Stimuli: negative emotional, sensory pain, affect pain, and neutral words. 4 tasks: conflicting color, negative emotional, sensory pain, and affect pain **.	Chronic pain group: high score on affective/evaluative and miscellaneous scales. Greater interference effect (chronic pain group): standard conflicting color Stroop.

Note: * Mean age and standard deviation of participating subjects not reported. ** Total duration of task not reported. **Abbreviations:** ACR = American College of Rheumatology; BOLD fMRI = Blood Oxygenation Level Dependent Functional Magnetic Resonance Imaging; BOLD = Blood Oxygenation Level Dependent; CLBP = Chronic Low Back Pain; CMSKP = Chronic Musculoskeletal Pain; CNP = Chronic Neuropathic Pain; ecStroop = emotional counting Stroop Task; EM = Episodic Migraine; FM = Frequent Migraine; fMRI = Functional Magnetic Resonance Imaging; FMS = Fibromyalgia Syndrome; HC = Healthy Controls; IHS = International Headache Society; HSF = High-Somatic Focus; LSF = Low-Somatic Focus; MBSR = Mindfulness-Based Stress Reduction; ncStroop = number counting Stroop Task; nStroop = neutral Stroop Task; NW = Neutral Words; RT = Reaction Time; SF = Symptom-related; TMD = Temporomandibular Disorder; WL = Waitlist.

## Data Availability

Not applicable.
